# Divergence in Single Kernel Characteristics and Grain Nutritional Profiles of Wheat Genetic Resource and Association Among Traits

**DOI:** 10.3389/fnut.2021.805446

**Published:** 2022-02-09

**Authors:** Anam Khalid, Amjad Hameed, Sadaf Shamim, Javed Ahmad

**Affiliations:** ^1^Department of Biological Sciences, Nuclear Institute for Agriculture and Biology, Pakistan Institute of Engineering and Applied Sciences, Islamabad, Pakistan; ^2^Wheat Research Institute, Ayub Agriculture Research Institute, Faisalabad, Pakistan

**Keywords:** wheat, single kernel characteristics, grain nutritional profile, cluster analysis, correlation, principal component analysis

## Abstract

*Triticum aestivum* is among the few species of crops which has been widely grown as a source of food. For seed quality trait analysis, wheat germplasm (77 genotypes) was collected from Pakistan's diverse agro-climatic regions. Significant variation (*p* < 0.05) was observed for tested parameters among tested genotypes. Genotypes with maximum protein content, i.e., GA2002 (16.5%) and Marvi (16.5%), moisture content, i.e., advance line 9,244 (11%), starch content, i.e., AARI 2011 (54.1%), zeleny sedimentation rate, i.e., advance line 2006 (44ml), wet gluten content, i.e., advance line 2006 (44%), kernel weight, i.e., advance line TC-4928 (41.6 ± 9.5 mg), kernel diameter, i.e., sassui (2.91 ± 0.32 mm), kernel moisture, i.e., AUQAB 2000 (11.7 ± 0.4%), Mairaj 2000 (11.7 ± 0.4%), and Barani-83 (11.7 ± 0.3%), and hardness index, i.e., Punjab 2011 (91 ± 39) are concluded as potential candidates to be explored for bakery products and the breeding program to improve quality attributes of wheat. Data were also analyzed for correlation, agglomerative hierarchical clustering, and principal component analysis (PCA). Cluster analysis clustered all genotypes into five different groups. The D^2^ statistics confirmed maximum diversity of cluster-V genotypes against genotypes of cluster-IV regarding single kernel characteristics, whereas cluster-II genotypes revealed maximum diversity against cluster-III genotypes relating to grain nutritional profile. The contribution of PC-I regarding single kernel characteristics toward variability was highest (48.58%) and revealed positive factor loadings for kernel weight, kernel diameter, and kernel moisture, while the contribution of PC-I with respect to grain nutritional profile toward variability was highest (59.76%) and showed positive factor loadings for moisture and starch content. Varieties having good quality attributes can be combined by breeders via various breeding methods with the aim of developing high quality wheat in the future.

## Introduction

Bread wheat (*Triticum aestivum*) is among the few species of crops that have been widely grown as a source of food, and it is considered of great significance primarily due to its grain which is comprised of proteins, carbohydrates, minerals (Cu, P, Mg, Fe, and Zn), and vitamins (niacin, thiamine, alpha tocopherol, and riboflavin). Among cereals, it is the most widely cultivated crop in the world, accounting for 17% of the world's crop acreage, supplying food to approximately 40% of the world population and providing 20% of the overall food calories besides protein in human diet. Approximately 3 billion people all over the world are facing malnutrition problems because of micronutrients deficiency (1, 2). Balanced food comprising sufficient micronutrients, proteins, and calories with few anti-nutritional constituents is required for appropriate development and growth (3).

Pakistan, Australia, United States, European Union, Turkey, Canada, India, China, Russia, and Ukraine are the main wheat producing countries, accounting for above 80% of wheat production throughout the world. Pakistan is the eighth biggest producer of wheat (4) contributing around 3.17% of the world wheat production from 3.72% of the wheat growing area (2). Commercial worth of bread wheat can be deduced from vital and principal characters for its superior end use quality (5).

Wheat quality is based upon composition of soil and environmental conditions like rainfall, temperature, and humidity (4). Genetic base is the main determinant for grain quality of wheat, and inheritance governing tools offer the basis for grouping genotypes with desirable genes (6). Therefore, it is necessary to understand the pattern of inheritance regarding the quality traits of wheat grain to upgrade the varieties on these aspects (6–8). Information about genetic divergence among adapted cultivars and elite breeding materials has substantial effect on the crop plants improvement, and this knowledge can be effectively utilized for the management of germplasm as well as selection of genotypes for diverse breeding purposes, though it is essential to determine whether various diversity assessment methods offer similar evidence relating to the degree of deviation among genotypes of wheat (9). Genetic divergence might be assessed through principal component as well as cluster analysis (5).

In Pakistan, breeding attempts stayed concentrated toward new varieties development with an improved yield potential and tolerance/resistance to rust diseases (2). During this former bidirectional breeding strategy the prospective aimed at grain quality improvement in wheat germplasm stayed fallow (6). Pakistani wheat varieties are grown over a wide agro-climatic range and as such are expected to exhibit yield and quality differences, so this makes it necessary to investigate the present status of wheat varieties available for food and nutritional purposes. In this view, a detailed study of genetic variability in nutritional status of diverse wheat genotypes was undertaken, followed by multivariate analysis which will provide valuable information regarding identification of superior accessions that can be utilized in wheat breeding programmes aimed at nutrition quality improvement.

## Materials and Methods

### Seed Material

Wheat germplasm of 77 varieties harvested during the year 2015 were collected from various centers within each agro-climatic zone of Pakistan, multiplied during 2 consecutive years, i.e., 2016 as well as 2017, and used to assess quality traits. Wheat varieties used in this study having year of release, source, and genotype pedigree have already been cited in Table 1 of our previously published paper Khalid and Hameed (10). The main objective was to access the status of quality traits of wheat, through analysis of genotypes evolved since the 1960s. Because selected genotypes hold great diversity, it includes genotypes from 1965 to 2011.

**Table 1:** Scale for categorization of wheat genotypes in low, medium, and high value for different quality attributes.

**Traits**	**Low**	**Genotypes**	**Medium**	**Genotypes**	**High**	**Genotypes**
**Single kernel characteristics**						
Kernel weight (mg)	29.9	GA2002, S-24, Raskoh-2005, C-591, 2006, Punjab-90, Khirman2006, Kohistan, C-228, Sarsabz, Pasban-90, AARI2011, Saleem 2000, Nia amber, millat 2011, Soghat90, Marvi, Nia sundar	30-32.9	Manthar 2003, SA-75, Bhakkar2000, Punjab-96, Mairaj 2000, Sindh-81, Sassui, Takbeer2000, Jauhar-78, Bakhtawar1993, Sitta, Nesser, Mehran-81, Bhittai2004, PARWAZ, SH-2002, Nia sunhari, Fareed 2006, Fakhare sarhad, Abadghar, NR-234, 2005, MEXI PAK, Margalla-99, WL-711, Pari-73, PAVON, BARS-2009, Punjab2011, IQBAL2000, 9021, 2156, Nifa- Bathoor, Sehar2006, 6544-6, Suleman, NR-421, Zardana, AUQAB2000, Tatara, Dharabi 2011	33-42	NARC400, 9244, AS-2002, Faisalabad2008, Galaxy 2013, Shafaq2006, UFAQ2002, Chakwal-50, Benazir-12, Lasani2008, Barani-83, Kiran 95, Inqulab91, Niea lalma 2013, Watan-94, V-8203, LU-26, TC-4928
Kernel diameter (mm)	NA		NA		NA	NA
Kernel moisture (%)	NA		NA		NA	NA
Hardness index (%)	69	Nifa- Bathoor, GA2002, Benazir-12, Niea lalma, 2013, LU-26, Takbeer2000, TC-4928, 2156	70–79	Inqulab91, Bhittai2004, Kiran95,9244, Punjab-96, Galaxy 2013, BARS200, Faisalabad2008, Sindh-81, Watan-94, Margalla-99, Sitta, AUQAB2000, Lasani2008, Sehar2006, AARI2011, V-8203, Barani-83, 9021, 6544-6, SH-2002, Mairaj 2000, Chakwal-50, Nia sunhari, Marvi, Kohistan, millat 2011, PAVON, UFAQ2002, AS-2002, Nesser, WL-711, Abadghar„ 2005, NR-234, Pari-73	80–91	Bhakkar2000, Fareed 2006, Jauhar-78, Sarsabz, Sassui, Nia sundar, Suleman, NARC400, Pasban-90, Nia amber, C-228, Shafaq2006, Bakhtawar1993, 2006, MEXI PAK, IQBAL2000, Tatara, Khirman2006, NR-421, Manthar 2003, Dharabi 2011, Fakhare sarhad, Soghat90, C-591, Raskoh-2005, Mehran-81, SA-75, PARWAZ, Punjab-90, S-24, Saleem 2000, Punjab2011
Grain Nutritional Profile						
Protein content (%)	NA		NA		NA	NA
Moisture content (%)	NA		NA		NA	NA
Starch content (%)	50.9	TC-4928, PARWAZ, Tatara, Dharabi 2011, 2006, Fakhare sarhad, Soghat90, millat 2011, Jauhar-78, NARC400, Niea lalma 2013, Marvi, AS-2002, Punjab2011, Sarsabz, C-228, Mehran-81, Sassui, Bhittai2004, GA2002, Raskoh-2005, Mairaj 2000, Sehar2006,	51–51.9	MEXI PAK, Pasban-90, Punjab-96, Lasani2008, V-8203, Bakhtawar1993, Suleman, Punjab90, PAVON, UFAQ2002, NR-234, Pari-73, IQBAL2000, Faisalabad2008, Khirman2006, Nia amber, Nia sunhari, Abadghar, Sitta, SA-75, S-24, Inqulab91, Watan-94,6544-6, NR-421, Fareed 2006, C591, Bhakkar 2000, Shafaq2006, Nia sundar, Kohistan, Zardana, AUQAB2000, Chakwal-50, SH-2002, Margalla-99,	52–54.1	BARS-2009, Galaxy 2013, Takbeer2000, Manthar 2003, Sindh-81, 2005, Nesser, Nifa- Bathoor, Kiran 95, Saleem 2000, WL-711,9244, LU-26,2156, 902, Benazir-12, Barani-83, AARI2011
Gluten content (%)	29	AARI2011, Barani-83, Galaxy 2013, Benazir-12, Sindh 81,9021,9244, NR-234, Nesser, AUQAB2000 Kiran 95, Nia sundar, Watan-94, Zardana	30–35.9	LU-26, Takbeer2000, Marvi, WL-711, PAVON, Bhakkar2000, Manthar 2003, Faisalabad2008 2156, Saleem 2000, SH-2002, Sehar2006, Bakhtawar1993, Jauhar-78, Khirman2006, NARC400,2005, Suleman, Raskoh-2005, Nifa- Bathoor, IQBAL2000, Shafaq2006, Lasani2008, millat2011, Punjab2011, Sassui, Nia amber, Margalla-99, SA-75, S-24, MEXI PAK, UFAQ2002, Fareed 2006, BARS-2009, V-8203, Nia sunhari, C-591, Kohistan, Sitta, Punjab-96, Chakwal-50, Niea lalma2013, Sarsaz, Bhittai2004, Abadghar, NR-421, Mehran-81, Pari-73	36–45	Fakhare sarhad, 6544-6, C-228, Punjab-90, Pasban-90, Soghat90, TC-4928, PARWAZ, Tatara, 2006
Zeleny (%)	69	9021, 9244, 2156, AARI2011, Nia sundar, Barani-83, 2005, Saleem 2000, Bhakkar2000, Manthar 2003, IQBAL2000, Shafaq2006, WL-711, PAVON, S-24, LU-26, AUQAB2000, Chakwal-50, Galaxy 2013, Benazir-12, SA-75, Nifa- Bathoor	70–79	Sehar2006, Sarsabz, Nesser, Sassui, Kohistan, NR-234, Zardana, Punjab-96, UFAQ2002, SH-2002, Fareed 2006, Khirman2006, Margalla-99 BARS-2009, Faisalabad2008, Bakhtawar1993, Watan-94, Abadghar, C-591, GA2002, Mairaj 2000, millat 2011, Fakhare sarhad, Takbeer2000, Marvi, AS-2002, Kiran 95, Bhittai2004, Suleman, Lasani2008, Punjab2011, V-8203, Niea lalma 2013, Jauhar-78, Sindh-81, Nia amber, Nia sunhari C-228, Pari-73, Dharabi 2011	80–86	MEXI PAK, Inqulab91, 6544-6, Sitta, Raskoh-2005, Punjab-90, Soghat90, NARC400, NR-421, Mehran-81, Pasban-90, TC-4928, PARWAZ, Tatara 2006

### Single Kernel Characterization

Tests were conducted at Cereals Laboratory, Ayub Agricultural Research Institute Faisalabad, for measuring grain physical characteristics through Single Kernel Characterization System (SKCS) which estimates the kernel's weight, diameter, moisture, and hardness index. The method involves the pouring of samples into an access hopper of the SKCS instrument which analyzed 300 kernels individually and recorded the results on a computer graph.

### Grain Nutritional Profile

Grain nutritional profile which includes starch, wet gluten, and protein content in addition to zeleny sedimentation rate was computed by Kernelyzer (Omeg Analyzer G model) in which cleaned sample (~600 g) was filled in a sample funnel and exposed to near infrared light of specific wavelength. Light penetrated into the sample molecules (partly absorbed and partly transmitted) which was then measured by a detection system. Amount of light absorbed by samples at different wavelength is directly proportional to concentration of chemical functional groups like C-H, N-H, and O-H. The explanation of these characteristic diatomic functional group frequencies lies in the approximately constant values of the stretching force constant of a bond in different molecules. Thus, the IR spectrum can be regarded as a “fingerprint” of the molecule (11).

### Statistical Analysis

All the data were taken in triplicate. Finally, data were subjected to analysis of variance, and genetic divergence was computed through cluster analysis by agglomerative hierarchical clustering and principal component analysis using computer software Microsoft Excel along with XLSTAT Version 2012.1.02, Copyright Addinsoft 1995-2012 (http://www.xlstat.com).

## Results

### Single Kernel Characteristics

#### Kernel Weight

Genotypes were divided into three groups based on comparative values of different studied parameters (Table 1). For kernel weight, measured by single kernel characterization system, significant difference was found among wheat genotypes, and this made it feasible to classify them in low, medium, and high classes. Eighteen genotypes were assembled in high class for kernel weight, in which the values ranged from 33 mg to 42 mg. Among these genotypes, highest kernel weight was found in TC-4928 (41.6 ± 9.5 mg). Forty-one genotypes with kernel weight ranging from 30–32.9% were grouped as the intermediate class. Eighteen genotypes were grouped in the third category that is of comparatively low kernel weight. In this class genotypes having kernel weight <29.9 mg were assembled. Among these genotypes with relatively low kernel weight, the least value was found in GA2002 (23.6 ± 9.1 mg).

#### Kernel Diameter

For kernel diameter, measured by single kernel characterization system, a low level of variation was found among wheat genotypes (Table 1) as values ranged from 2.59 to 2.91 mm. Among these genotypes, highest kernel diameter was found in Sassui (2.91 ± 0.32 mm) while the lowest value was observed in S-24 (2.59 ± 0.34 mm).

#### Kernel Moisture

For kernel moisture, a little variation was found among wheat genotypes (Table 1) and values ranged from 10.6 to 11.7%. Among tested genotypes, highest kernel moisture was found in AUQAB 2000 (11.7 ± 0.4%), Mairaj 2000 (11.7 ± 0.4%), and Barani-83 (11.7 ± 0.3%), while the lowest value was observed in Pasban-90 (10.6 ± 0.3%).

#### Hardness Index (HI)

For hardness index, considerable difference was observed among wheat genotypes, and this made it feasible to classify them in low, medium, and high classes (Table 1). Thirty-two genotypes were assembled in high category for hardness index, in which the values ranged from 80 to 91. Among these genotypes hardness index was found highest in Punjab 2011 (91 ± 39). Thirty-seven genotypes with hardness index ranging from 70 to 79 were grouped as intermediate class. Eight genotypes were grouped in the third category that is of comparatively low hardness index. In this class, genotypes having hardness index <69 were grouped. Among these genotypes with relatively low kernel hardness; the least value was found in Nifa-Bathoor (47 ± 17).

### Grain Nutritional Profile

#### Protein Content

For protein content in wheat seeds measured by near infrared spectroscopy using Omeg Analyzer, no significant variation was found among wheat genotypes (Table 1); however, values ranged from 11.9 to 16.5%. Among these genotypes, maximum protein content was observed in genotypes GA2002 (16.5%) and Marvi (16.5%) but the lowest value was observed in Inqulab-91 (11.9%).

#### Moisture Content

Moisture content was found to be same in all genotypes (Table 1). Moisture content ranged from 10 to 11% with the highest in advance line 9,244 (11%) and lowest in genotype Bhittai2004 (10%).

#### Starch Content

Starch content varied significantly among wheat genotypes (Table 1) and this made it likely to classify the in low, medium, and high classes. Eighteen genotypes were grouped in high category for starch content, where the values ranged from 52 to 54.1%. Among these genotypes, maximum starch content was observed in AARI 2011 (54.1%). Thirty-six genotypes with starch content ranging from 51 to 51.9% were classified as intermediate class. Twenty-three genotypes were grouped in the third category that is of comparatively low starch content. In this class, genotypes with starch content <50.9% were assembled. Among these genotypes with relatively low starch content, the least value was found in TC-4928 (48.4%).

#### Wet Gluten Content

Wet gluten content in wheat seeds was observed variable among wheat genotypes (Table 1) so this made it feasible to classify them in low, medium, and high classes. Fifteen genotypes were assembled in the high category for wet gluten content, where the values varied from 36 to 45%. Among these genotypes, highest wet gluten content was found in advance line 2006 (44%). Forty-eight genotypes with wet gluten content ranging from 30 to 35.9% were grouped as the intermediate class. Fourteen genotypes were grouped in the third category which is of comparatively low wet gluten content. In this class, genotypes having wet gluten content <29% were grouped. Among these genotypes with relatively low wet gluten content, the least value was observed in AARI2011 (24%).

#### Zeleny Sedimentation Rate

For Zeleny sedimentation rate in wheat seeds, significant difference was observed among wheat genotypes, and this made it feasible to classify them in low, medium, and high classes (Table 1). Fifteen genotypes were grouped in high category for zeleny sedimentation rate, where the values varied from 80 to 86 ml. Among these genotypes, highest zeleny sedimentation rate was found in advance line 2006 (86 ml). Forty genotypes with zeleny sedimentation rate ranging from 70 to 79 ml were grouped as intermediate class. Twenty-two genotypes were assembled in the third category which is of relatively low zeleny sedimentation rate. In this category, genotypes with zeleny sedimentation rate <69 mL were assembled. Among these genotypes having relatively low zeleny sedimentation rate, the least value was found in 9021 (58 mL).

#### Correlations/Associations Patterns Among Traits

Simple correlation coefficient values revealed significant relationships which makes course of selection more effective to plan breeding approach (Table 2).

**Table 2:** Correlation matrix among different quality parameters in wheat genotypes.

**Variables**	**Protein content (%)**	**Moisture content (%)**	**Starch content (%)**	**Wet gluten content (%)**	**Zeleny (ml)**	**Hardness index**	**Kernel weight (mg)**	**Kernel moisture (%)**	**Kernel diameter (mm)**
Protein content (%)	**1**								
Moisture content (%)	**−0.326**	**1**							
Starch content (%)	**−0.541**	**0.257**	**1**						
Wet gluten content (%)	**0.453**	**−0.231**	**−0.743**	**1**					
Zeleny (ml)	**0.400**	**−0.403**	**−0.727**	**0.697**	**1**				
Hardness Index	0.064	**–**0.177	**−0.299**	**0.241**	0.162	**1**			
Kernel Weight (mg)	**–**0.112	**0.229**	0.044	**–**0.120	0.006	**−0.276**	**1**		
Kernel Moisture (%)	**–**0.192	**0.365**	0.108	**–**0.085	**−0.279**	**–**0.143	0.214	**1**	
Kernel diameter (mm)	0.085	0.026	**–**0.101	0.005	0.145	**–**0.160	**0.769**	0.084	**1**

*Bold values shows significant positive and negative correlation among traits*.

Protein content showed significant positive association with gluten content and zeleny sedimentation rate, though it has significant negative correlation with moisture and starch content. Moisture content showed significant positive correlation with starch content, kernel weight, and kernel moisture whereas it showed significant negative correlation with protein, wet gluten, and zeleny sedimentation rate. Significantly positive correlation was revealed by starch content with moisture content. However, it had significantly negative association with protein content, wet gluten content, zeleny sedimentation rate, and hardness index. Wet gluten content showed significant positive association with protein content, zeleny sedimentation rate, and hardness index while it showed negative association with moisture and starch content. Zeleny sedimentation rate had significant positive correlation with protein and wet gluten content while it had negative correlation with moisture, starch, and kernel moisture. Hardness index had significant positive association with wet gluten content whereas it had negative association with starch content and kernel weight. Kernel weight showed positive correlation with moisture content as well as kernel diameter but had negative correlation with hardness index. Kernel moisture showed significant association with moisture content and negative association with zeleny sedimentation rate. Kernel diameter had significant positive association with kernel weight.

### Cluster Analysis

#### Single Kernel Characteristics

**Figure 1:**
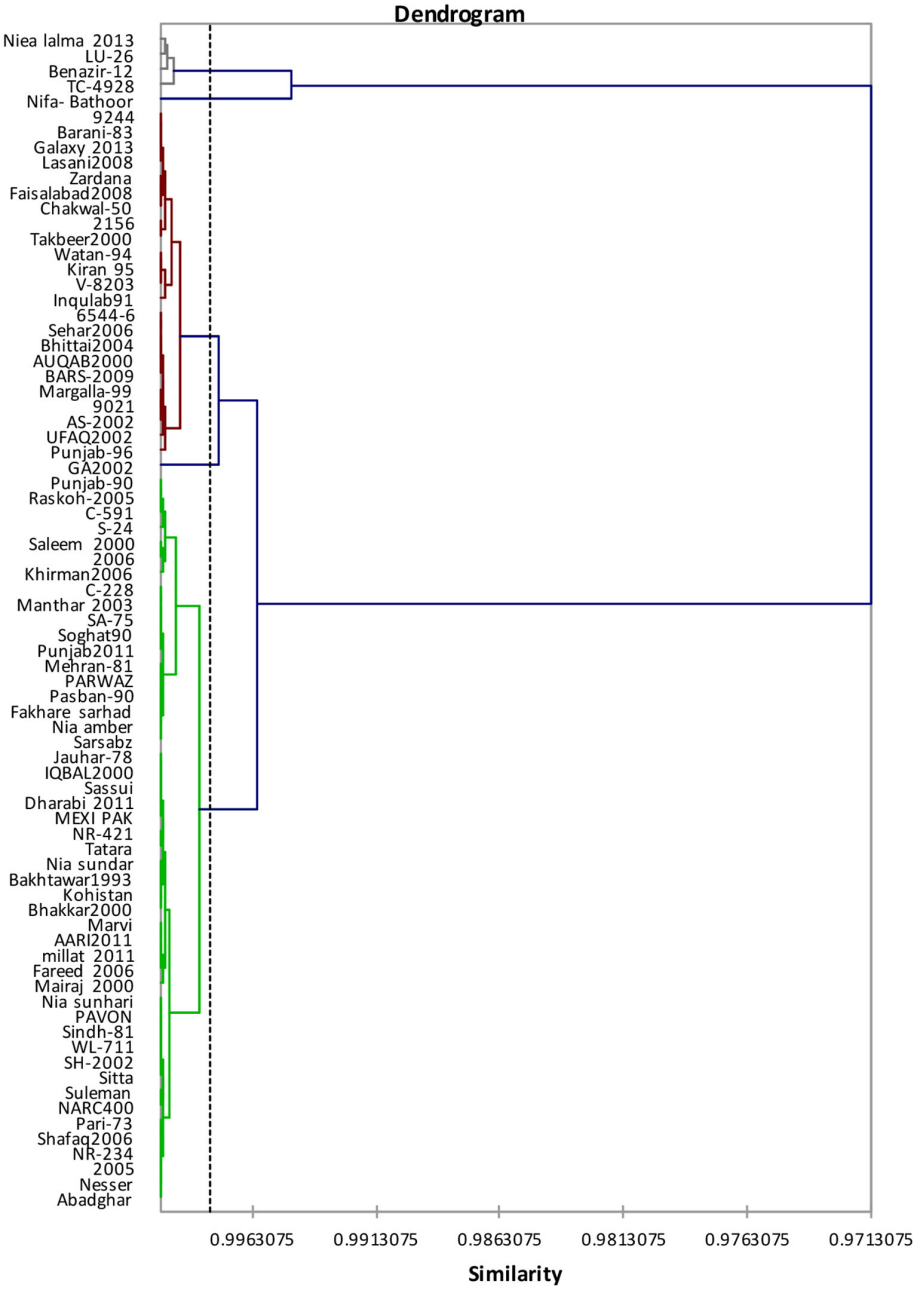
Tree diagram based on four traits regarding single characteristics for different wheat genotypes.

Genotypes clustering on the basis of studied traits are shown in Figure 1. Cluster analysis assembled 77 genotypes of wheat into five clusters as shown in Table 3. Cluster-I encompassed 48 genotypes superseded by 4 and 23 genotypes correspondingly in cluster-II and cluster-III.

**Table 3:** Distribution of wheat genotypes regarding single kernel characteristics in different clusters.

**Cluster**	**Genotypes**
I	PAVON, PARWAZ, MEXI PAK, IQBAL2000, Bhakkar2000, SH-2002, Manthar 2003, Fareed 2006, Shafaq2006, Mairaj 2000, Dharabi 2011, AARI2011millat 2011, Punjab2011, Bakhtawar1993, Tatara, Fakhare sarhad, Jauhar-78, Sindh-81, Sarsabz, Soghat90, Sassui, Khirman2006, Nia amber, Nia sunhari, Nia sundar, NARC400, Marvi, Abadghar, C-228, C-591, 2006, 2005, Kohistan, Suleman, NR-234, NR-421, Sitta, Nesser, Raskoh-2005, Mehran-81, Saleem 2000, Punjab-90, SA-75, Pasban-90, S-24, Pari-73, WL-711
II	LU-26, Niea lalma 2013, Benazir-12, TC-4928
III	AUQAB2000, UFAQ2002, AS-2002, Lasani2008, Inqulab91, Sehar2006, Chakwal-50, BARS-2009, Faisalabad2008 Galaxy 2013, V-8203, Takbeer2000, Kiran 95, Bhittai2004, Barani-83, Watan-94, 2156, 9021, 6544-6, 9244, Margalla-99, Zardana, Punjab-96
IV	GA2002
V	Nifa-Bathoor

GA-2002 and Nifa-bathoor has been eluded because it plunges in cluster-IV and cluster-V (Table 4). Although cluster analysis assembled genotypes together with larger phenotypic similarity, the groups had not necessarily included all the genotypes from identical origin.

**Table 4:** Mean values of different quality traits regarding single kernel characteristics of wheat genotypes in cluster analysis.

**Clusters**	**Hardness index**	**Kernel weight (mg)**	**Kernel moisture (%)**	**Kernel diameter (mm)**
1	81.542	30.540	11.148	2.715
2	62.000	37.100	11.275	2.843
3	73.522	33.017	11.248	2.764
4	49.000	23.600	11.200	2.630
5	47.000	32.500	11.500	2.690

Cluster-I was comprised of genotypes with large hardness index. Cluster-II was comprised of genotypes with higher kernel weight and kernel diameter; however, the cluster-V genotypes possessed greater kernel moisture (Table 4). Pairwise Mahalanobis distances (D^2^ statistics) are given in Table 5. Cluster-V genotypes revealed maximal divergence against cluster-IV genotypes, whereas least variances were detected between cluster-II as well as cluster-III due to minimum amount of genetic divergence.

**Table 5:** D2 statistics among different clusters regarding grain nutritional profile.

	**Cluster I**	**Cluster II**	**Cluster III**	**Cluster IV**	**Cluster V**
Cluster I	0				
Cluster II	19.676	0			
Cluster III	9.886	10.164	0		
Cluster IV	7.024	26.513	16.531	0	
Cluster V	6.965	16.413	6.566	11.799	0

#### Grain Nutritional Profile

**Figure 2:**
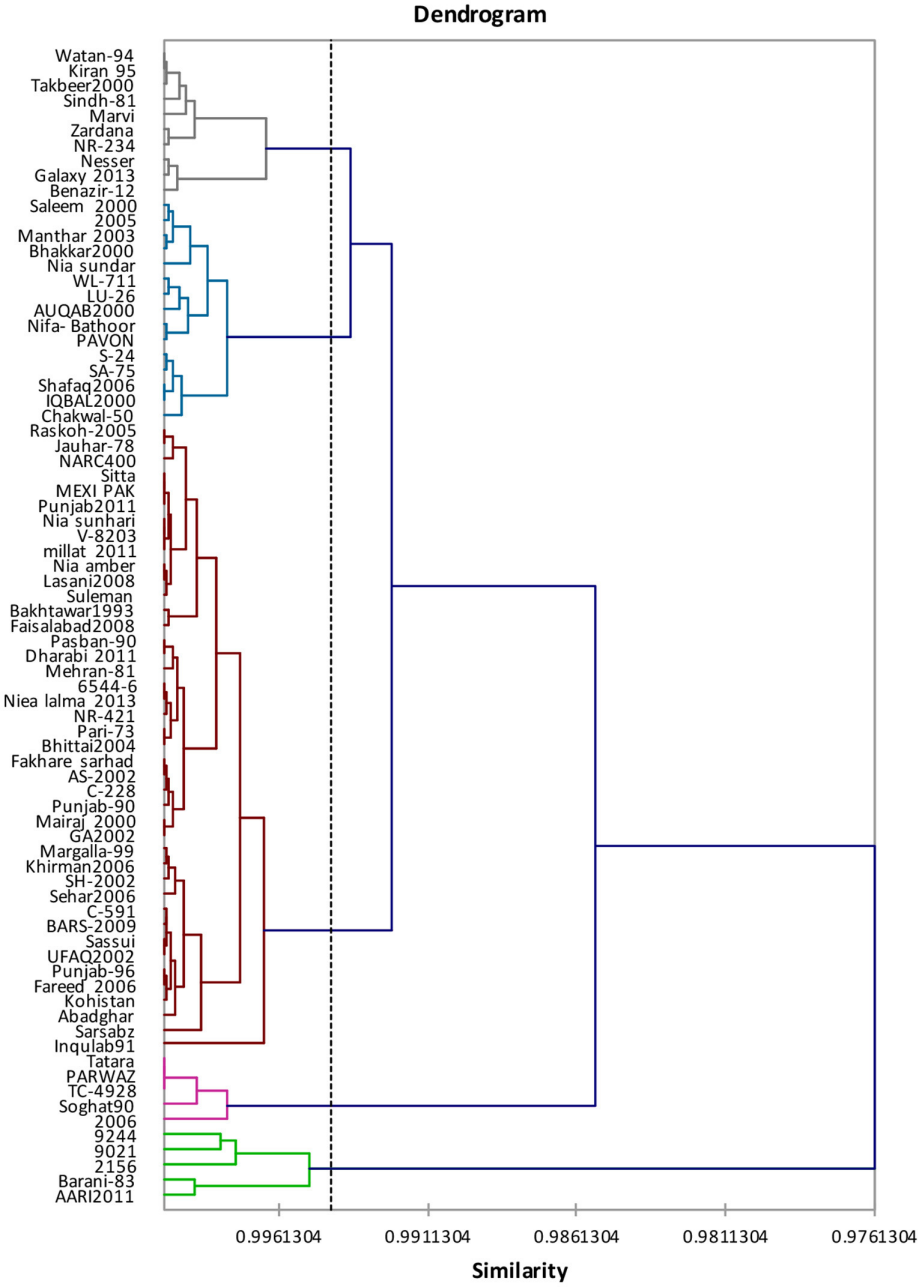
Tree diagram based on four traits regarding single characterization system for different wheat genotypes.

Genotypes clustering on the basis of examined traits are shown in Figure 2. Cluster analysis clustered 77 genotypes of wheat into five clusters as shown in Table 6. Cluster-I included 15 genotypes superseded by 42, 5, 10, and 5 genotypes correspondingly in cluster-II cluster-III, cluster-IV, and cluster-V.

**Table 6:** Distribution of wheat genotypes regarding grain nutritional profile in different clusters.

**Cluster**	**Genotypes**
I	PAVON, LU-26, IQBAL2000, AUQAB2000, Bhakkar2000, Manthar 2003, Shafaq2006, Chakwal-50, Nia sundar, 2005, Saleem 2000, SA-75, Nifa- Bathoor, S-24, WL-711
II	PARWAZ, Tatara, Soghat90, 2006, TC-4928
III	MEXI PAK, UFAQ2002, GA2002, SH-2002, AS-2002, Fareed 2006, Mairaj 2000, Lasani2008, Inqulab91, Sehar2006, BARS-2009, Dharabi 2011, Faisalabad2008, millat 2011, Punjab2011, V-8203, Bakhtawar1993, Fakhare sarhad, Niea lalma 2013 Jauhar-78, Sarsabz, Bhittai2004, Sassui, Khirman2006, Nia amber, Nia sunhari, NARC400, Abadghar, C-228, C-591, Kohistan 6544-6, Suleman, NR-421, Margalla-99, Sitta, Raskoh-2005, Mehran-81, Punjab-90, Pasban-90, Punjab-96, Pari-73
IV	AARI2011, Barani-83, 2156, 9021, 9244
V	Galaxy 2013, Takbeer2000, Sindh-81, Kiran 95, Benazir-12, Watan-94, Marvi, NR-234, Nesser, Zardana

Although cluster analysis assembled genotypes together with larger phenotypic similarity, the groups had not necessarily included all the genotypes from identical origin. Cluster-II was comprised of genotypes with higher protein content, wet gluten content, and zeleny sedimentation rate, while Cluster-IV was comprised of genotypes with maximum moisture content and starch content (Table 7). Pairwise Mahalanobis distances (D^2^ statistics) are shown in Table 8 where cluster-II genotypes revealed ultimate diversity against cluster-III genotypes, whereas low divergence was detected between cluster-IV and cluster-V due to minimum value of genetic divergence.

**Table 7:** Mean values of different quality traits regarding grain nutritional profile of wheat genotypes in cluster analysis.

**Clusters**	**Protein content (%)**	**Moisture content (%)**	**Starch content (%)**	**Wet gluten content (%)**	**Zeleny (mL)**
1	15.620	10.520	51.913	31.533	67.133
2	16.060	10.360	49.320	40.200	84.600
3	15.938	10.407	50.938	34.119	76.619
4	14.280	10.700	53.320	27.400	61.800
5	15.440	10.420	51.900	28.500	73.400

**Table 8:** D2 statistics among different clusters regarding grain nutritional profile.

	**Cluster I**	**Cluster II**	**Cluster III**	**Cluster IV**	**Cluster V**
Cluster I	0				
Cluster II	20.614	0			
Cluster III	8.395	12.224	0		
Cluster IV	33.274	18.743	26.268	0	
Cluster V	34.599	15.692	26.528	9.127	0

### Principal Component Analysis

#### Single Kernel Characteristics

The data showed that out of 4 principal components (PCs), only one viz. PC-1 had Eigen values >1 and contributed 48.579% of total combined variability among diverse genotypes (Table 9). The rest of three PCs elucidated minor (non-significant) variation; hence, they were not worth construing. The contribution of PC-I toward variability was highest (48.58%). The PC-I showed positive factor loadings for kernel weight, kernel diameter, and kernel moisture while negative factor loading for hardness index.

**Table 9:** Principal component analysis for different quality parameters regarding single kernel characteristics in wheat genotypes.

	**F1**	**F2**	**F3**	**F4**
Eigenvalue	1.943	0.996	0.848	0.213
Variability (%)	48.579	24.899	21.190	5.332
Cumulative %	48.579	73.478	94.668	100.000
Eigen vector: variables				
Hardness index	−0.337	−0.477	0.806	0.097
Kernel weight (mg)	0.661	−0.181	0.082	0.723
Kernel moisture (%)	0.256	0.769	0.576	−0.107
Kernel diameter (mm)	0.619	−0.385	0.113	−0.675

**Figure 3:**
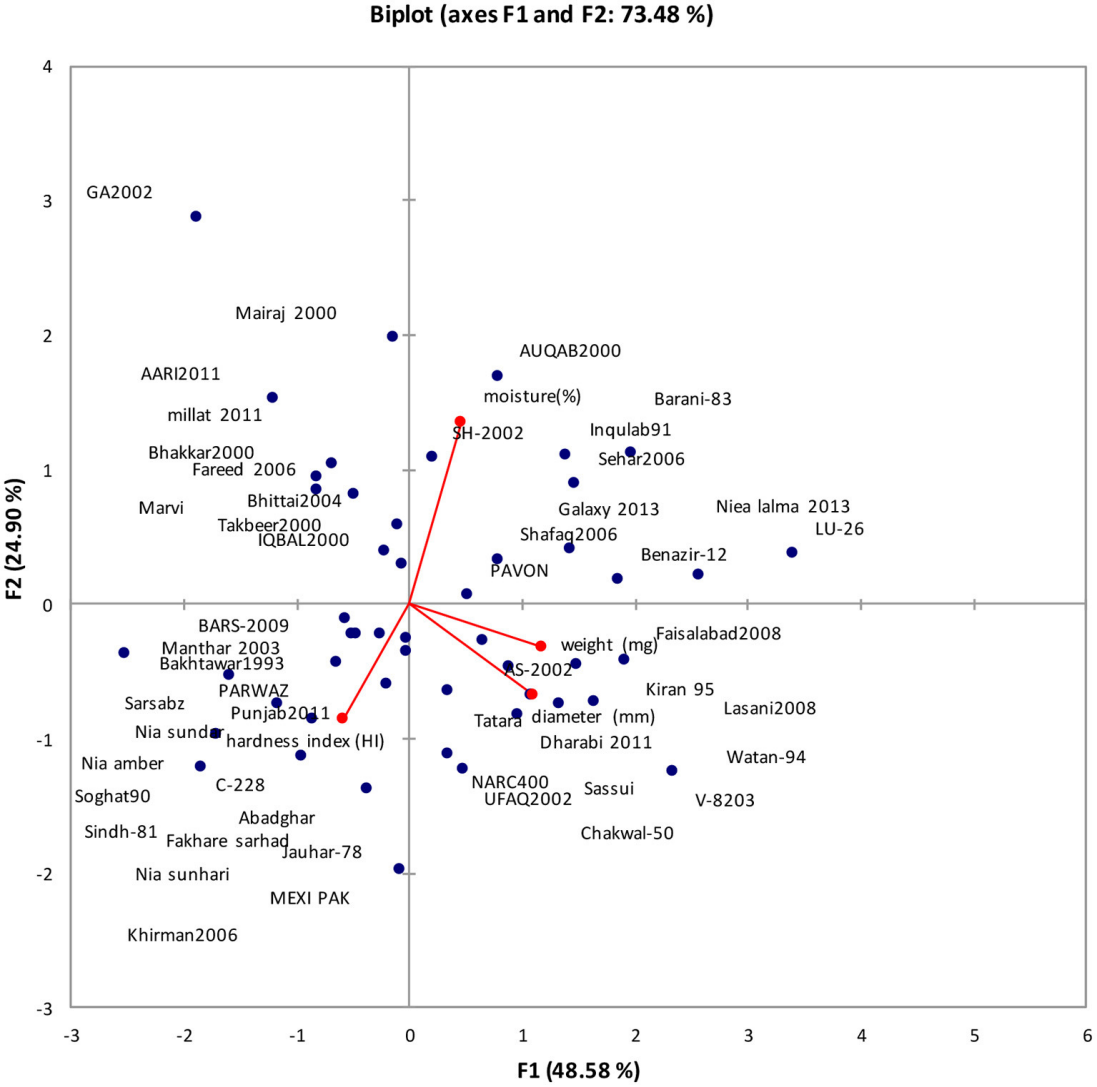
Bi-plot of wheat genotypes for first two principal components regarding single kernel characterization system.

The first two principal components who contributed 73.48% toward total variance were plotted on PC-I x-axis and PC-II on y-axis to find out the association between different clusters (Figure 3). The biplot shows the association of 77 wheat genotypes for 4 traits. The genotype by trait (G-T) biplot with respect to mean performance of the wheat genotypes described the 73.48% of the total variation of the consistent data. In this bi-plot, a vector is haggard from source to each quality attribute that facilitates the conception of inter-relationships among traits. The trait's vector length enables the verification of the magnitude of its influence (12) on quality. It can be seen that weight was positively correlated with diameter while hardness index was negatively correlated with all other traits.

#### Grain Nutritional Profile

The data revealed, out of 5 principal components (PCs), only one viz. PC-1 possessed eigen values >1 and accounted for 59.76% of overall combined variation among diverse genotypes (Table 10). The rest of the four PCs elucidated minor (non-significant) variation and hence were not worth construing. The contribution of PC-I toward variability was highest (59.76%). The PC-I showed positive factor loadings for moisture and starch content while negative factor loading for wet gluten content, zeleny sedimentation rate, and protein content.

**Table 10:** Principal component analysis for different quality parameters regarding grain nutritional profile in wheat genotypes.

	**F1**	**F2**	**F3**	**F4**	**F5**
Eigenvalue	2.988	0.872	0.643	0.274	0.223
Variability (%)	59.757	17.437	12.868	5.474	4.464
Cumulative %	59.757	77.193	90.062	95.536	100.000
Eigen vector:					
variables					
Protein content (%)	−0.400	−0.155	0.870	−0.071	0.232
Moisture content (%)	0.291	0.894	0.233	−0.179	0.170
Starch content (%)	0.513	−0.263	0.003	0.259	0.775
Wet Gluten content (%)	−0.492	0.321	−0.154	0.774	0.177
Zeleny (mL)	−0.500	0.058	−0.407	−0.545	0.534

**Figure 4:**
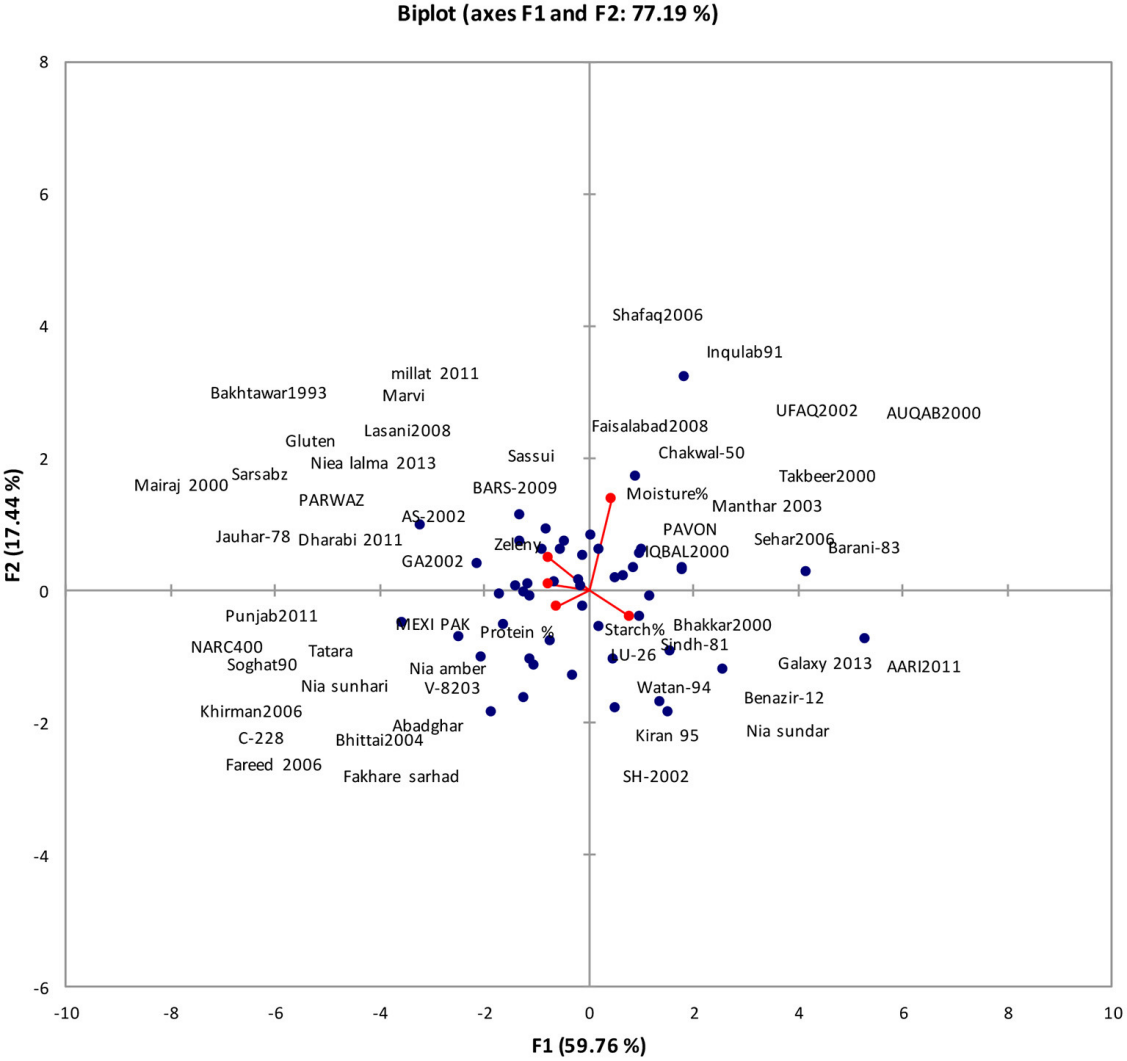
Bi-plot of wheat genotypes for first two principal components regarding grain nutritional profile.

The first two principal components who contributed 77.19% to total variance were presented on PC-I (x-axis) and PC-II (y-axis) to find out the linkage between various clusters (Figure 4). This biplot showed the association of 77 wheat genotypes for 5 traits. It can be seen that moisture content was found higher in 11 genotypes while starch content was higher in 10 genotypes, whereas zeleny sedimentation rate, protein content, and wet gluten content are negatively correlated with all other traits.

## Discussion

Grain protein percentage is the main constituent of grain quality (2, 13). It defines the quality, seedling vigor, grain yield, and nutritional status of wheat flour (14, 15). Difference in protein composition and concentration remarkably alters the bread making quality (15). Weather, genetic makeup, and prevailing growth conditions are chief aspects affecting protein content (4). Kernel of wheat comprises of 8 to 17% proteins which is usually found in the endosperm (2). Various proteins in the mature kernel have either structural or metabolic part, yet the majority of proteins found in kernel are storage proteins, which plays a vital part for germinating the embryo in the form of nitrogen reserves (13). Protein content determination is of great importance as it is the main element in grading, blending, and storage confirmation in the course of loading (2). In present study, highest protein content was found in GA2002 and Marvi (16.5%). Previously, the highest mean value for protein using near infrared spectroscopy and Kjeldahl methods was 10.27% for genotype Gandam-711. Another study reported that wheat genotypes contained 9.15~10.27% protein (2). Further in a study, genotype HD-2687 revealed highest value of total protein content (12.83%) during the grain nutritional quality characterization (3, 4) reported the highest crude protein content of (12.45%) in Inqalab-91. In another study on wheat genotypes of Sindh, Pakistan, highest protein content was observed in genotypes Anmol (15.42%), TJ-83 (15.2%), and Kiran-95 (14.60%) (13). The overall comparison revealed that protein content in the present study was generally in the previously reported range. However, for the first time we reported that two tested genotypes, i.e., GA2002 and Marvi, had 16.5% protein and can be utilized for improving this trait in wheat.

Moisture content is defined as amount of water stored in each wheat variety and is of utmost importance with respect to productivity (16). In the developmental stage of the plant, moisture plays a crucial role in the starch production and ensures endosperm filling out, ensuing optimal milling. Elevated moisture content augments proteolytic in addition to lipolytic activities causing nutrients loss. It is an indicator of grain storability as well and may forecast profit margins in milling. Flour with less moisture content is more stable in the period of storage. Moisture content >14.5% fascinates bacteria, insects, and molds (4). Hence, it does not directly influence grain quality, although it can decrease grain's storage period with moisture content higher than suggested (4). In the current study, moisture content was found highest in advance line 9,244 (11%) while highest kernel moisture was found in three genotypes, i.e., AUQAB 2000 (11.7 ± 0.4%), Mairaj 2000, (11.7 ± 0.4%) and Barani-83 (11.7±0.3%), whereas in previous studies, highest level of moisture was shown by genotype Wafaq-01 (9.27%) (2) and (3.547 g per 100 grain) in the same variety during the study of physiochemical traits of wheat genotypes in same or diverse ecological conditions of Pakistan (16). In another study, moisture content of 10.23% has been reported in Inqalab-91 (4).

Starch is a principal component of most cereals and wheat is among them (17). It provides most of the nutrients and enormous amount of energy in human diet and is also a source of palatability. It sustains the flour viscosity to enhance the dough extensibility. It is an essential aspect for bakery items (3) and used to impart textural characteristics as well. It is also employed as thickening, moisture retention, adhesion, gelling, stabilizing, and film forming ingredients besides preservative to sustain softness and moisture in bakery items (17). In our study, highest starch content was found in AARI 2011 (54.1%). Previously, highest starch content was found in genotype HD-2687 (74.30%) during the assessment of grain nutritional quality (3).

Zeleny sedimentation value defines the flour's sedimentation level dispersed in lactic acid solution in a standard time interval. Sedimentation rate of flour's suspension relies on the composition as well as content of wheat protein, hardness, pan, in addition to hearth loaves volume and is affected by swelling of gluten fraction. Greater content of gluten gives rise to slower sedimentation and elevated value of Zeleny test (18). This test is comparatively economical, less time-consuming, requires less labor, cheap, and needs no extensive laboratory apparatus (19). It is a highly consistent technique for assessing quality besides quantity of wheat protein and hence is a better indicator of baking quality and bread making strength in wheat (18–20). The highest zeleny sedimentation rate was found in advance line 2006 (86 ml) in this study. However, in an earlier study, Inqalab-91 revealed higher SDS sedimentation test average value (30.32 mL) (4). In another study, Hruskova and Famera (18) assessed 318 wheat varieties for Zeleny sedimentation value using near infrared technique and reported a range from 17 to 66 mL. Moreover, maximum rate of zeleny sedimentation rate in varieties C- 518 and GA 2002 and a range of 50.67–80.34 ml has been reported (21). Though studies have been undertaken to describe the zeleny sedimentation rate in wheat genotypes, outcomes comparison is not likely possible in the majority of cases due to different procedures, besides distinct solvents and evaluating techniques.

Wheat genetic structure is responsible for affecting qualitative features of gluten like wheat flour strength and gluten content (4). Gluten is a form of protein composed of two major kinds of subunit monomeric gliadin plus polymeric glutinins. Both are the seed storage proteins constituting 75–85% of entire grain protein. They don't have any enzymatic purpose; however, these proteins are technically active and can help in the dough development, manufacturing of baked foodstuffs like chapatti, cookies, cake, and bread. The difference in concentration of glutenin affects chapatti making (13). In current study, advance line 2006 (44%) showed highest wet gluten content while the lowest was recorded in AARI 2011 (24%).

Kernel weight is an indicator of grain size as well as possible flour quantity in the kernel (22). In our study, maximum kernel weight was detected in advance line TC-4928 (41.6 ± 9.5 mg) and kernel diameter was found highest in genotype Sassui (2.91 ± 0.32 mm). The endosperm texture can be stated as a degree of the resistance to distortion or deformation (22). Grain hardness/strength is a significant grain quality trait which has an important part in the cereal grains processing besides the end-use quality of cereal grain foodstuffs like breads and snacks (23). Grain hardness is also responsible for plant protection against molds and insect attack (24). In the present study, hardness index was found to be maximum in genotype Punjab 2011 (91 ± 39).

Correlation studies give an idea about the nature and extent of relationship between any two sets of metric/quantitative characters. An unusually high correlation factor between two quality traits proposes a robust heritable relationship and perhaps a fine genetic base (25). From this, it might be likely to generate genetic advancement in one trait by selecting the other pair (26). In the present study, significantly positive correlation was revealed by protein content with wet gluten and zeleny sedimentation rate. Starch content displayed highly significant and positive correlation with moisture content. Wet gluten content had significantly positive correlation with protein content, zeleny sedimentation rate, and hardness index. Protein content and wet gluten content revealed significant positive correlation with zeleny sedimentation rate. Significantly positive correlation was shown by hardness index with wet gluten content. Kernel moisture and kernel diameter showed significant positive correlation with kernel weight. Kernel moisture had significantly positive association with moisture content. Kernel diameter showed significantly positive correlation with kernel weight. Hence, the study of associations among these qualitative traits can be useful for the selection of superior wheat genotypes having a group of desired characters by the breeders in the breeding program.

Clustering (multivariate analysis) allows the combination of genotypes/traits information to categorize any population into core groups on the basis of similarities (27). Cluster analysis might be considered as a potent tool to classify germplasm, providing consistent basis in the base material selection to design breeding tactics in the future. Cluster analysis grouped 77 wheat genotypes with respect to single kernel characteristics into 5 clusters. According to Pairwise Mahalanobis distances (D^2^ statistics), cluster-V genotypes depicted utmost divergence against cluster-IV genotypes whereas, cluster analysis regarding grain nutritional profile grouped 77 wheat genotypes into 5 clusters and Pairwise Mahalanobis distances (D^2^ statistics) showed that the cluster-II genotypes revealed maximum variability against cluster-III genotypes.

It is obvious from present findings that cluster analysis might be considered as an effective tool to catalog germplasm which offers consistent foundation in the base material selection to design breeding tactics in the future (28, 29). However, the authors believe that one should be careful of breeding practices and genetic constrictions to obtain likely genetic improvement for desired characters while making base material choices. Outcomes of the current study showed that multivariate analysis assists in placing genotypes in numerous clusters depending upon PC(s) values.

Principal component analysis is a traditional method employed for examining data, compression, as well as visualization of data set features (30). In fact, it reveals the verification of main contributors to the entire variability at every differentiation axis. Eigen values assist in identifying the impeding factor and their total is almost equivalent to overall number of variables (5). The contribution of PC-I regarding single kernel characteristics toward variability was highest (48.58%). It revealed positive factor loadings for kernel weight, kernel diameter, and kernel moisture while the contribution of PC-I regarding grain nutritional profile toward variability was highest (59.76%), and it showed positive factor loadings for moisture and starch content.

In the present study, varieties like Punjab 2011, UQAB 2000, ARRI 2011 depicted maximum value for various quality parameters and assigned a good chapatti making quality, while Ujala-16 has very good chapatti making quality, whereas Barani-83 has fair chapatti making quality. Similar grading for these wheat varieties is also available previously (https://aari.punjab.gov.pk/crop_varieties_wheat). However, for the first time we reported here that GA-2002, Marvi, and sassui hold very good chapatti making quality.

Considerable variation for various traits has been revealed by divergence analysis of collected germplasm. The wide range of diversity in phenotypic characters has proved to be an effective tool in classifying germplasm. The acquired information can be of great interest to wheat breeders willing to evolve new varieties with desired nutritional qualities.

## Conclusion

Wheat genotypes from diverse geographic regions of Pakistan were found to have enormous diversity in terms of quality attributes. Based on the current report, genotypes with maximum protein (GA2002 and Marvi), moisture (advance line9244), starch content (AARI 2011), zeleny sedimentation rate and wet gluten content (advance line 2006), kernel weight (advance line TC-4928), kernel diameter (sassui), kernel moisture (AUQAB 2000, Mairaj 2000 and Barani−83), and hardness index (Punjab 2011) are potential candidates for improving end use quality for these traits. Further, genotypes of cluster-V showed maximum diversity against genotypes of cluster-IV with respect to single kernel characteristics. Principal component analysis revealed positive factor loadings for kernel weight, kernel diameter, and kernel moisture relating to single kernel characteristics. On the basis of these novel outcomes, superior accessions can be utilized in wheat breeding programmes aimed at nutrition quality improvement. Additionally, findings will also help to encounter the challenge of world malnourishment.

## Data Availability Statement

The original contributions presented in the study are included in the article/supplementary material, further inquiries can be directed to the corresponding author.

## Author Contributions

AK: overall execution of the experiment, analytical work, collection of data after seeds quality traits analysis, organization of resulting data, write up and revision of manuscript. AH: planning, design and finalization of basic idea of experiment and overall supervision during analytical work, statistical analysis of data using XL-STAT software, presentation of resulting data in the form of graphs and revision and finalization of manuscript. SS and JA helped in carrying out experiments at Ayub Agricultural Research Institute, Faisalabad. All authors contributed to the article and approved the submitted version.

## Conflict of Interest

The authors declare that the research was conducted in the absence of any commercial or financial relationships that could be construed as a potential conflict of interest.

## Publisher's Note

All claims expressed in this article are solely those of the authors and do not necessarily represent those of their affiliated organizations, or those of the publisher, the editors and the reviewers. Any product that may be evaluated in this article, or claim that may be made by its manufacturer, is not guaranteed or endorsed by the publisher.
